# Supplementary material for: Mining the heparinome for cryptic antimicrobial peptides that selectively kill Gram-negative bacteria
Source: Mol Syst Biol. 2025 May 23;21(7):889–910. doi: 10.1038/s44320-025-00120-6 (PMC12223310; doi:10.1038/s44320-025-00120-6)
Supplement: Supplementary file 1 — Appendix [file 44320_2025_120_MOESM1_ESM.pdf]

Appendix for

Mining the heparinome for cryptic antimicrobial peptides that selectively kill Gram-negative bacteria

Roberto Bello-Madruga[1,#], Daniel Sandín[1,#], Javier Valle[2], Jordi Gómez[1], Laura Comas[3], María Nieves Larrosa[4,5], Juan José González-López[4,5], María Ángeles Jiménez[3], David Andreu[2,*] and Marc Torrent[1,*]

[1] Systems Biology of Infection Laboratory, Department of Biochemistry and Molecular Biology, Universitat Autònoma de Barcelona. Cerdanyola del Vallès, 08193, Spain [2] Department of Medicine and Life Sciences, Universitat Pompeu Fabra, Barcelona Biomedical Research Park. 08003 Barcelona, Spain. [3] Departamento de Química-Física Biológica, Instituto de Química Física Rocasolano (IQFR-CSIC). Serrano 119, 28006, Madrid, Spain. [4] Servei de Microbiologia, Hospital Universitari Vall d'Hebron. Barcelona, 08035, Spain [5] Dr. M.N. Larrosa, Dr. J.J. González-López. Departament de Genètica i Microbiologia, Universitat Autònoma de Barcelona. Cerdanyola del Vallès, 08193, Spain.

* Correspondence to: David Andreu (david.andreu@upf.edu) and Marc Torrent (marc.torrent@uab.cat)
# Both authors contributed equally to this work

Table of Content

Appendix Figures 3

Appendix Figure S1. Electrostatic potential surfaces of HBPs. 3

Appendix Figure S2. NMR chemical shift of HBP-5 in presence of heparin disaccharide. 4

Appendix Figure S3. Molecular dynamics simulation of HBP-5 4

Appendix Figure S4. HPLC-MS analysis 5

Appendix Figure S5. HPLC-MS analysis 6

Appendix Figure S6. HPLC-MS analysis 7

Appendix Figure S7. HPLC-MS analysis 8

Appendix Tables 9

Appendix Table S1. Additional data for peptides HBP-1 to HBP-5. 9

Appendix Table S2. MIC and MBC values (µM) of all peptides against gram-negative clinical isolates 10

Appendix Table S3. Hemolytic and cytotoxic activities of peptides. 10

Appendix Table S4. EC_{50} and $t_{1/2}$ values for HBP peptides and LL-37 10

Appendix Table S5. Calculated secondary structure percentages by circular dichroism in 5 mM PB 11

Appendix Table S6. Calculated secondary structure percentages by circular dichroism in 10 mM SDS 11

Appendix Table S7. Calculated secondary structure percentages by circular dichroism in heparin 20 µg/mL 11

Appendix Table S8. Calculated secondary structure percentages by circular dichroism in LPS 50 µg/mL 11

Appendix Table S9. NMR analyses 12

Appendix Table S10. Summary of NMR structural statistic parameters calculated for HBP-5 in DPC micelles. 12

Appendix Table S11. Sequences and molecular mass of HBP-5 mutants 12

Appendix Table S12. *In vivo* study. Exact p values corresponding to statistical analysis 13

Appendix Figures

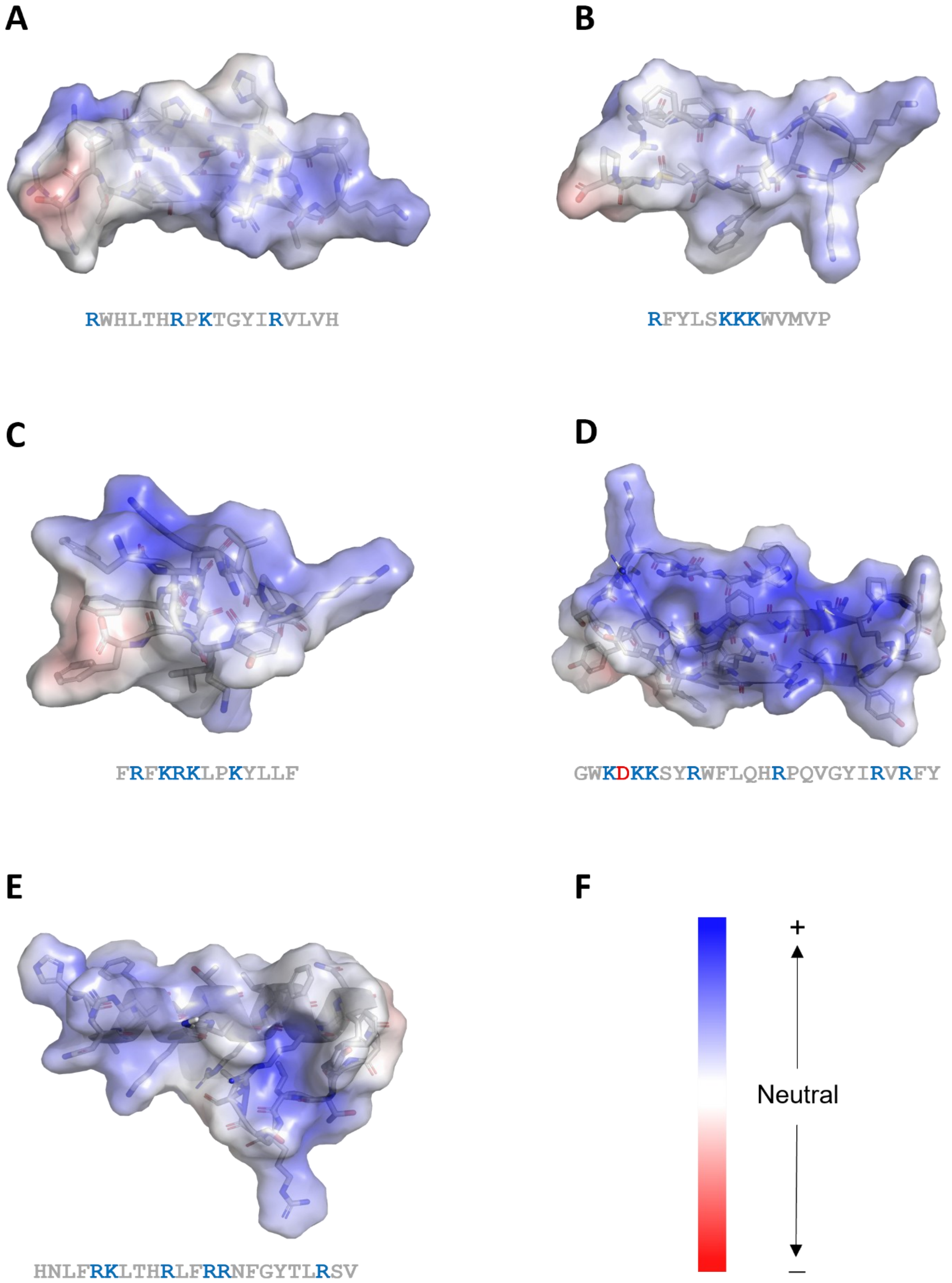

Appendix Figure S1. Electrostatic potential surfaces of HBPs. Global electrostatic potential surfaces of predicted **(A)** HBP-1, **(B)** HBP-2, **(C)** HBP-3, **(D)** HBP-4 and **(E)** HBP-5 models. **(F)** Electrostatic potentials for negative, positive, and neutral regions are shown in red, blue and grey, respectively.

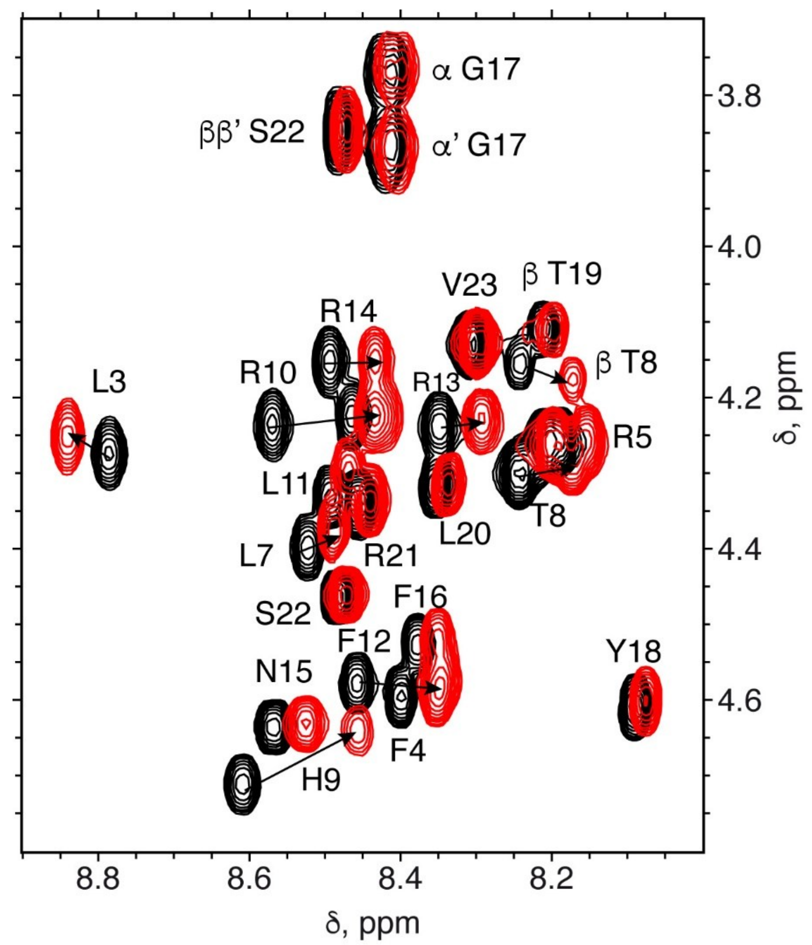

Appendix Figure S2. NMR chemical shift of HBP-5 in presence of heparin disaccharide. Overlay of selected regions of 2D $^{1}H,^{1}H$ TOCSY spectra for free **HBP-5** (black contours) and for **HBP-5** in the presence of heparin disaccharide HIS at 1:1 ratio (red contours). In both cases, aqueous solution at pH 5.5 and 5 ºC. Cross-peaks between a and HN protons are labelled, and the arrows connect peaks corresponding to free and H1S-bound **HBP-5**.

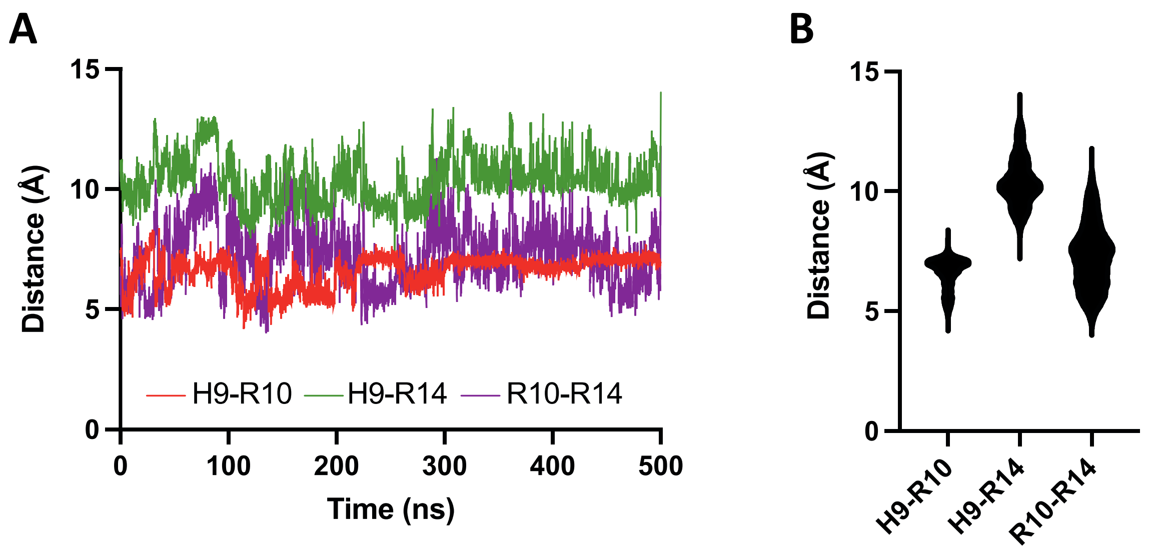

Appendix Figure S3. Molecular dynamics simulation of HBP-5. (A) Distance between residues H9 and R10 (red), H9 and R14 (green), and R10 and R14 (purple) during the simulation. **(B)** Average distances for each residue pair over the simulation.

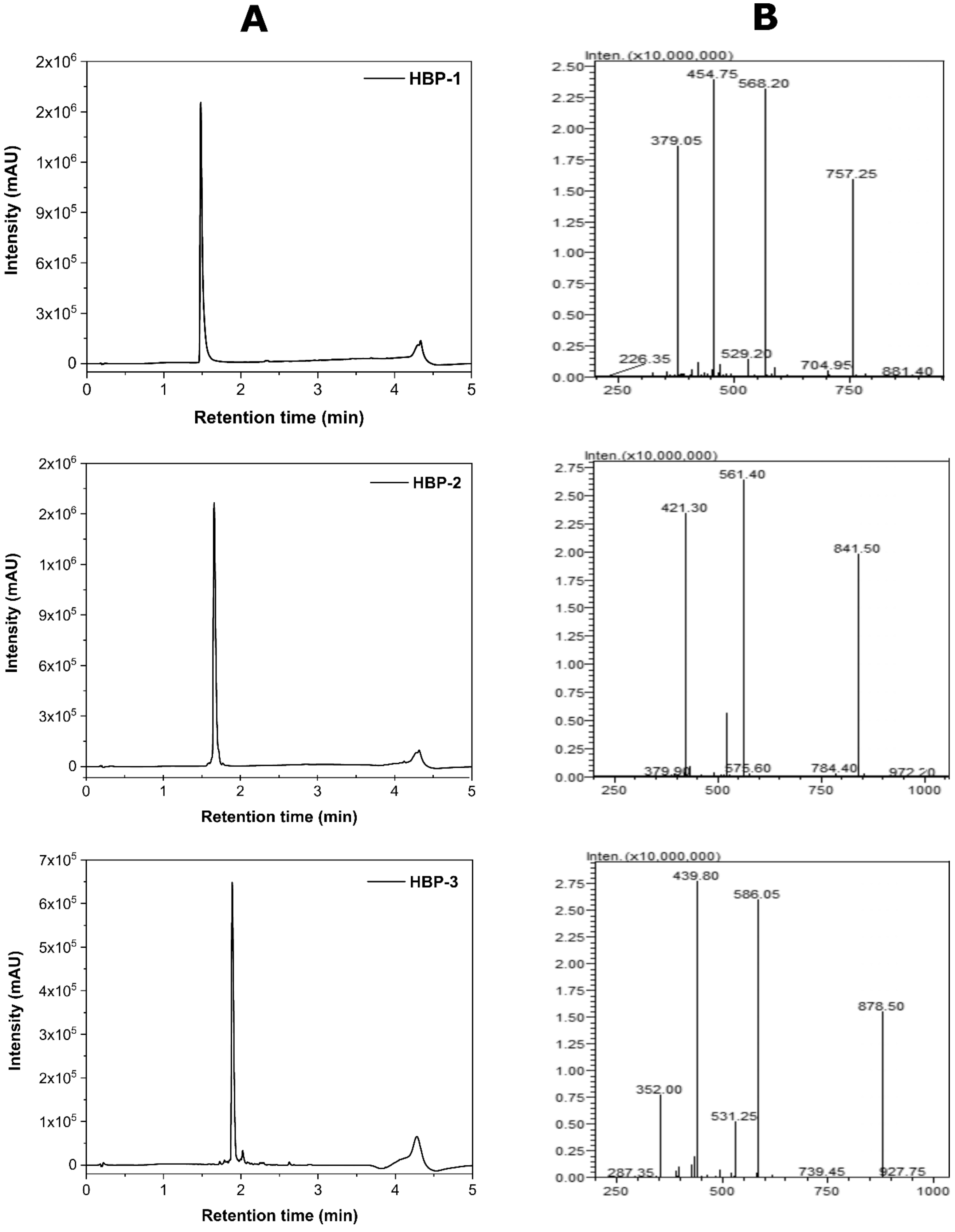

Appendix Figure S4. HPLC-MS analysis. HPLC profiles **(A)** and MS spectra **(B)** of purified peptides.

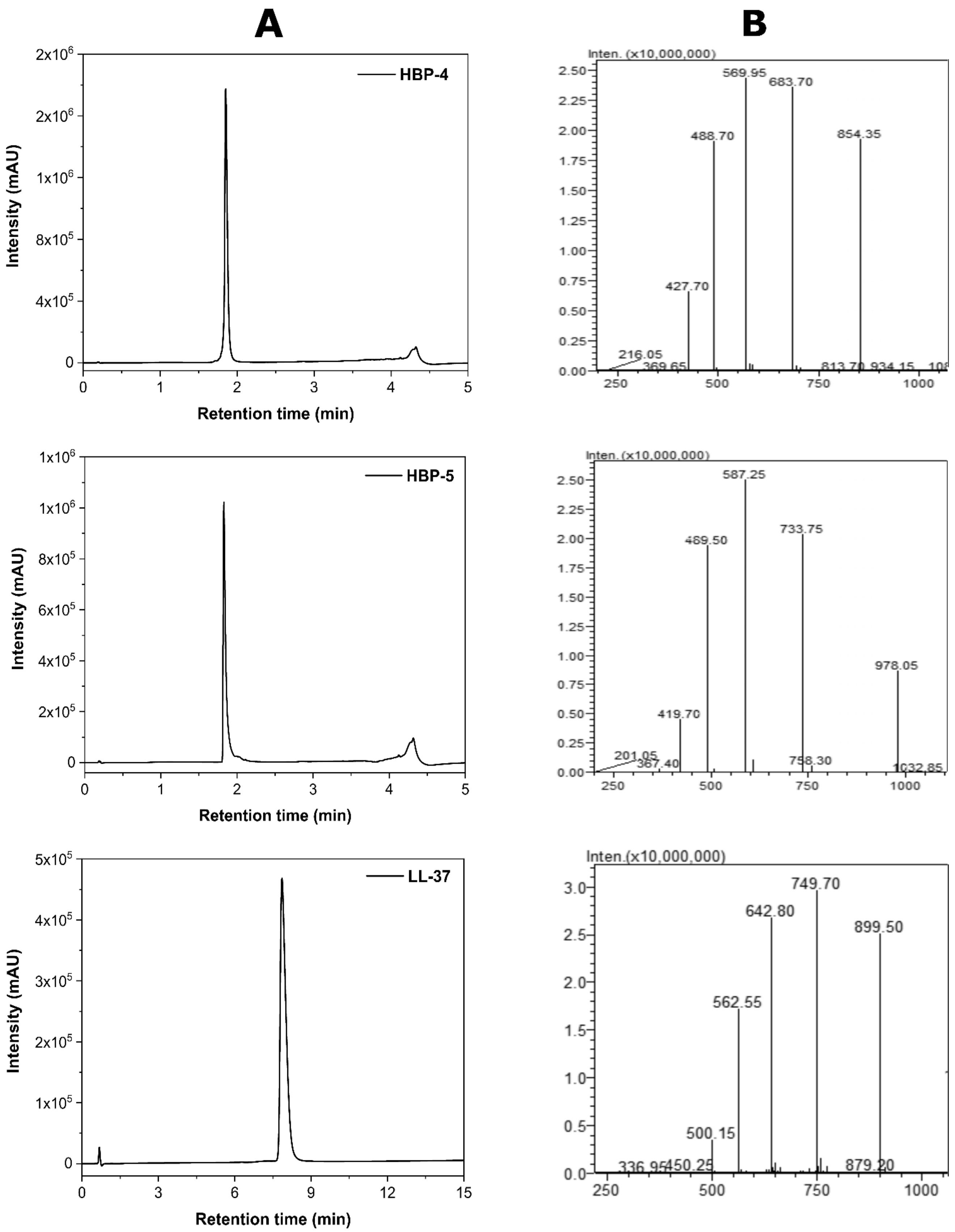

Appendix Figure S5. HPLC-MS analysis. HPLC profiles **(A)** and MS spectra **(B)** of purified peptides

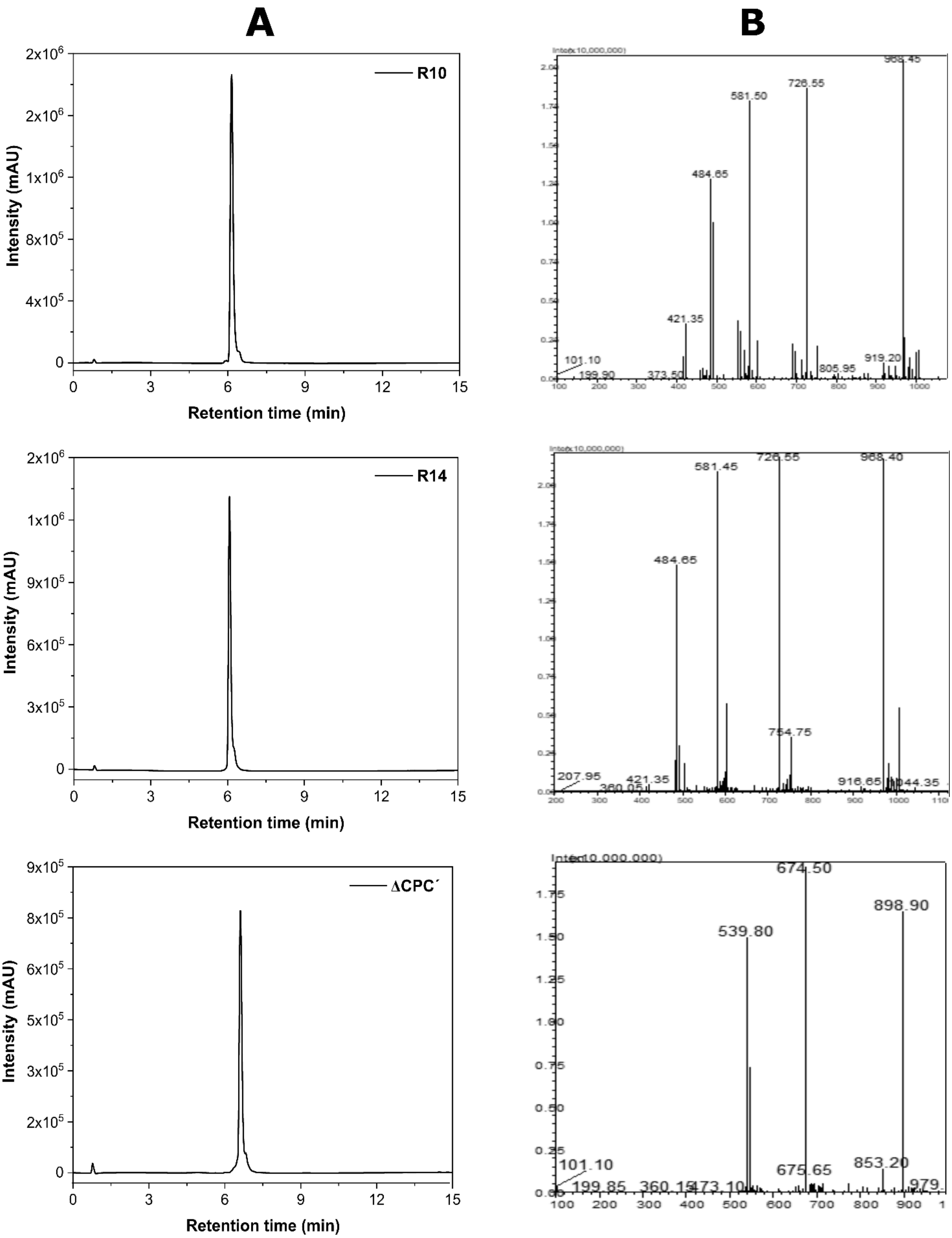

Appendix Figure S6. HPLC-MS analysis. HPLC profiles **(A)** and MS spectra **(B)** of purified peptides

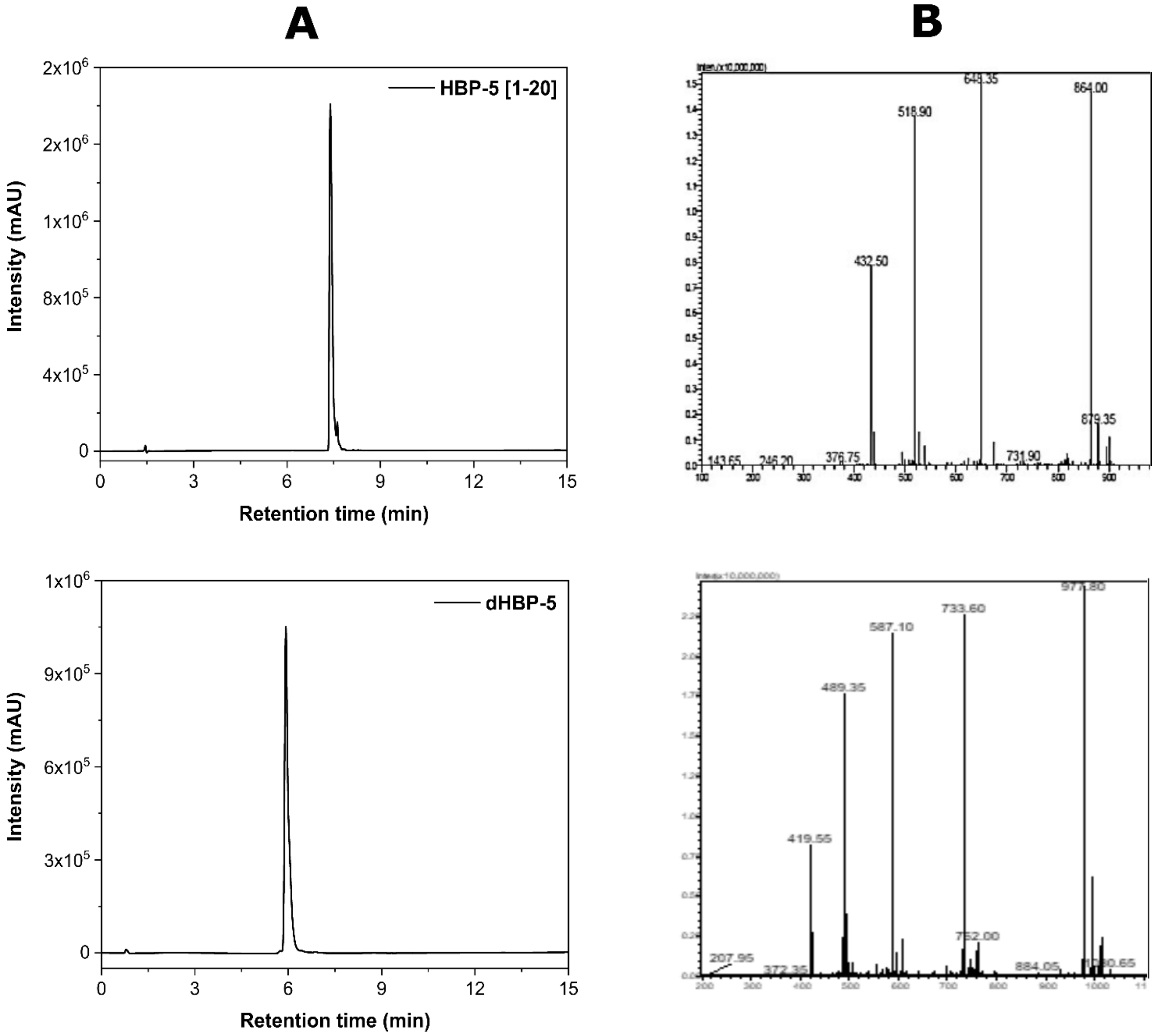

Appendix Figure S7. HPLC-MS analysis. HPLC profiles **(A)** and MS spectra **(B)** of purified peptides

Appendix Tables

Appendix Table S1. Additional data for peptides HBP-1 to HBP-5.

Peptide	Sequence	Source Protein	Protein GO Biological processes
HBP-1	RWHLTHRPKTGYIRVLVH	Thrombospondin-2 (P35442)	• Cell adhesion<br>• Negative regulation of angiogenesis<br>• Positive regulation of synapse assembly
HBP-2	RFYLSKKKWVMVP	Alpha-1-antichymotrypsin (ACT, P01011)	• Acute-phase response<br>• Inflammatory response<br>• Maintenance of gastrointestinal epithelium<br>• Negative regulation of endopeptidase activity<br>• Regulation of lipid metabolic process
HBP-3	FRFKRKLPKYLLF	Amiloride-sensitive amine oxidase (P19801)	• Amine metabolic process<br>• Cellular response to azide<br>• Cellular response to copper ion<br>• Cellular response to heparin<br>• Cellular response to histamine<br>• Putrescine metabolic process<br>• Response to antibiotic
HBP-4	GWKDKKSYRWFLQHRPQVGYIRVRFY	Cartilage oligomeric matrix protein (P49747)	• Animal organ morphogenesis<br>• Animal organ senescence<br>• Apoptotic process<br>• Artery morphogenesis<br>• BMP signaling pathway<br>• Bone mineralization<br>• Cartilage homeostasis<br>• Chondrocyte development<br>• Chondrocyte proliferation<br>• Collagen fibril organization<br>• Growth plate cartilage development<br>• Limb development<br>• Multicellular organism aging<br>• Multicellular organism growth<br>• Musculoskeletal movement<br>• Negative regulation of apoptotic process<br>• Negative regulation of hemostasis<br>• Platelet aggregation<br>• Positive regulation of chondrocyte proliferation<br>• Protein homooligomerization<br>• Protein processing<br>• Protein secretion<br>• Regulation of bone mineralization<br>• Regulation of gene expression<br>• Response to unfolded protein<br>• Skeletal system development<br>• Skin development<br>• Tendon development<br>• Vascular associated smooth muscle cell development<br>• Vascular associated smooth muscle contraction
HBP-5	HNLFRKLTHRLFRRNFGYTLRSV	Heparin cofactor 2 (P05546)	• Blood coagulation<br>• Chemotaxis<br>• Negative regulation of endopeptidase activity

[a] UniProt database codes added in brackets.

Appendix Table S2. MIC and MBC values (µM) of all peptides against gram-negative clinical isolates.

MIC / MBC (µM)						
Peptide	*E. coli* CFT073	*E. coli* 1166795	*P. aeruginosa* 827651	*P. aeruginosa* 827632	*A. baumannii* 3878	*A. baumannii* 3880
HBP-1	3.1 / 3.1	1.6 / 1.6	25 / 50	25 / 25	1.6 / 1.6	12.5 / 12.5
HBP-2	50 / 100	12.5 / 12.5	>100 / >100	>100 / >100	50 / 50	>50 / >50
HBP-3	12.5 / 25	1.6 / 1.6	12.5 / 12.5	25 / 25	6.3 / 6.3	6.3 / 6.3
HBP-4	0.8 / 1.6	<0.1 / <0.1	3.1 / 6.3	6.3 / 6.3	0.8 / 0.8	1.6 / 1.6
HBP-5	0.4 / 0.8	<0.1 / <0.1	0.8 / 1.6	1.6 / 1.6	0.2 / 0.2	0.4 / 0.4

Appendix Table S3. Hemolytic and cytotoxic activities of peptides.

Peptide	Hemolysis (%)[a]	LC_{50} (MRC-5 cells, µM)	LC_{50} (HepG2 cells, µM)
HBP-1	9.2 ± 0.6	>200	>200
HBP-2	4.0 ± 1.0	>200	>200
HBP-3	8.0 ± 1.0	>200	>200
HBP-4	31.7 ± 0.1	35 ± 1	38 ± 13
HBP-5	23.5 ± 0.2	69 ± 2	80 ± 7
LL-37	64.3 ± 0.7	26 ± 3	53 ± 2

[a] Hemolysis data was assayed at a peptide concentration of 250 µM.

Appendix Table S4. EC_{50} and $t_{1/2}$ values for HBP peptides and LL-37 (Figures 2A and 2C respectively).

Peptide	LPS Affinity EC_{50} (µM)	DiSC3(5) $t_{1/2}$ (s)
HBP-1	0.50 ± 0.10	38 ± 8
HBP-2	0.25 ± 0.60	53 ± 12
HBP-3	0.65 ± 0.50	25 ± 5
HBP-4	0.85 ± 0.60	37 ± 3
HBP-5	0.98 ± 0.70	34 ± 2
LL-37	0.95 ± 0.80	58 ± 3

Appendix Table S5. Calculated secondary structure percentages by circular dichroism in 5 mM PB using CDSSTR in DichroWeb (http://dichroweb.cryst.bbk.ac.uk/html/process.shtml).

Peptide	α-Helix	β-Strand	Turns	Unordered	Total
HBP-1	0.07	0.33	0.24	0.35	0.99
HBP-2	0.04	0.32	0.24	0.37	0.97
HBP-3	0.09	0.31	0.26	0.34	1
HBP-4	0.06	0.36	0.23	0.34	0.99
HBP-5	0.12	0.27	0.26	0.34	0.99

Appendix Table S6. Calculated secondary structure percentages by circular dichroism in 10 mM SDS using CDSSTR in DichroWeb (http://dichroweb.cryst.bbk.ac.uk/html/process.shtml).

Peptide	α-Helix	β-Strand	Turns	Unordered	Total
HBP-1	0.07	0.39	0.23	0.32	1.01
HBP-2	0.10	0.38	0.23	0.30	1.01
HBP-3	0.11	0.35	0.25	0.30	1.01
HBP-4	0.05	0.38	0.23	0.33	0.99
HBP-5	0.50	0.24	0.15	0.19	1

Appendix Table S7. Calculated secondary structure percentages by circular dichroism in heparin 20 µg/mL using CDSSTR in DichroWeb (http://dichroweb.cryst.bbk.ac.uk/html/process.shtml).

Peptide	α-Helix	β-Strand	Turns	Unordered	Total
HBP-1	0.04	0.45	0.22	0.29	1
HBP-2	0.02	0.43	0.22	0.32	0.99
HBP-3	0.06	0.46	0.19	0.28	0.99
HBP-4	0.06	0.42	0.21	0.31	1
HBP-5	0.37	0.23	0.17	0.23	1

Appendix Table S8. Calculated secondary structure percentages by circular dichroism in LPS 50 µg/mL using CDSSTR in DichroWeb (http://dichroweb.cryst.bbk.ac.uk/html/process.shtml).

Peptide	α-Helix	β-Strand	Turns	Unordered	Total
HBP-1	0.02	0.39	0.23	0.35	0.99
HBP-2	0.01	0.39	0.21	0.36	0.97
HBP-3	0.06	0.38	0.21	0.33	0.98
HBP-4	0.06	0.44	0.2	0.3	1
HBP-5	0.2	0.27	0.25	0.29	1.01

Appendix Table S9. NMR analyses. Averaged $\Delta\delta_{H\alpha}$ and $\Delta\delta_{C\alpha}$ values for **HBP-5** in aqueous solution at pH 5.0 and in DPC micelles (30 mM DPC) at pH 5.0 at 25 ºC. The percentage of helical structure was estimated from these values. [a] Errors are reported as the standard deviation.

HBP-5						
Conditions	Helical residues	$\Delta\delta_{H\alpha}$, ppm	% helix from $\Delta\delta_{H\alpha}$	$\Delta\delta_{C\alpha}$, ppm	% helix from $\Delta\delta_{C\alpha}$,	Averaged % helix[a]
Aqueous solution	3-11	-0.039	10	+0.02	5	5±5
DPC micelles	3-11	-0.307	79	+2.95	96	85±9

Appendix Table S10. Summary of NMR structural statistic parameters for the ensemble of the 20 lowest target function conformers calculated for **HBP-5** in DPC micelles.

HBP-5 in DPC micelles	
Number of distance restraints	
Intraresidue & sequential (i – j ≤ 1)	198
Medium range (1 < |i – j| < 5)	37
Long range (|i – j| ≥ 5)	3
Total number	238
Averaged total number per residue	10.3
Number of dihedral angle constraints	
Number of restricted ϕ angles	21
Number of restricted ψ angles	20
Total number	41
Pairwise RMSD (Å)	
All residues	2-22
Backbone atoms	1.6±0.7
All heavy atoms	2.6±0.7
N-terminal helix	3-15
Backbone atoms	0.3±0.2
All heavy atoms	1.6±0.3
Ramachandran plot (%)	
Most favoured regions	93.8
Additionally allowed regions	6.2
Generously allowed regions	0.0
Disallowed regions	0.0

Appendix Table S11. Sequences and molecular mass of HBP-5 mutants

Peptide	Sequence [a]	Molecular mass (Da)	
		Theory	Found
R10	HNLFRKLTH**Q**LFRRNFGYTLRSV-NH_2	2903.3	2902.5
R13	HNLFRKLTHRLF**Q**RNFGYTLRSV-NH_2	2903.4	2902.5
R14	HNLFRKLTHRLFR**Q**NFGYTLRSV-NH_2	2903.4	2902.5
CPC	HNLFRKLT**AA**LFR**A**NFGYTLRSV-NH_2	2695.1	2694.3

[a] The amino acids highlighted in blue and red correspond to the CPC motif and the mutated amino acids, respectively.

Appendix Table S12. *In vivo* study. Exact p values corresponding to statistical analysis

		p values of two-way ANOVA analysis			
		Liver	Spleen	Lung	Kidney
Male	Vehicle vs. HBP-5	$< 1.0 \times 10^{-15}$	1.5×10^{-15}	$< 1.0 \times 10^{-15}$	$< 1.0 \times 10^{-15}$
	Vehicle vs. HBP-5 [1-20]	$< 1.0 \times 10^{-15}$	9.0×10^{-15}	$< 1.0 \times 10^{-15}$	$< 1.0 \times 10^{-15}$
	Vehicle vs. dHBP-5	$< 1.0 \times 10^{-15}$	4.0×10^{-15}	$< 1.0 \times 10^{-15}$	$< 1.0 \times 10^{-15}$
	Vehicle vs. LL-37	$< 1.0 \times 10^{-15}$	5.5×10^{-15}	4.0×10^{-15}	$< 1.0 \times 10^{-15}$
Female	Vehicle vs. HBP-5	2.0×10^{-15}	$< 1.0 \times 10^{-15}$	$< 1.0 \times 10^{-15}$	$< 1.0 \times 10^{-15}$
	Vehicle vs. HBP-5 [1-20]	1.9×10^{-15}	$< 1.0 \times 10^{-15}$	$< 1.0 \times 10^{-15}$	$< 1.0 \times 10^{-15}$
	Vehicle vs. dHBP-5	2.0×10^{-15}	$< 1.0 \times 10^{-15}$	$< 1.0 \times 10^{-15}$	$< 1.0 \times 10^{-15}$
	Vehicle vs. LL-37	4.0×10^{-15}	$< 1.0 \times 10^{-15}$	$< 1.0 \times 10^{-15}$	$< 1.0 \times 10^{-15}$
