# Supplementary figures and images for: Mining the heparinome for cryptic antimicrobial peptides that selectively kill Gram-negative bacteria
Source: Mol Syst Biol. 2025 May 23;21(7):889–910. doi: 10.1038/s44320-025-00120-6 (PMC12223310; doi:10.1038/s44320-025-00120-6)

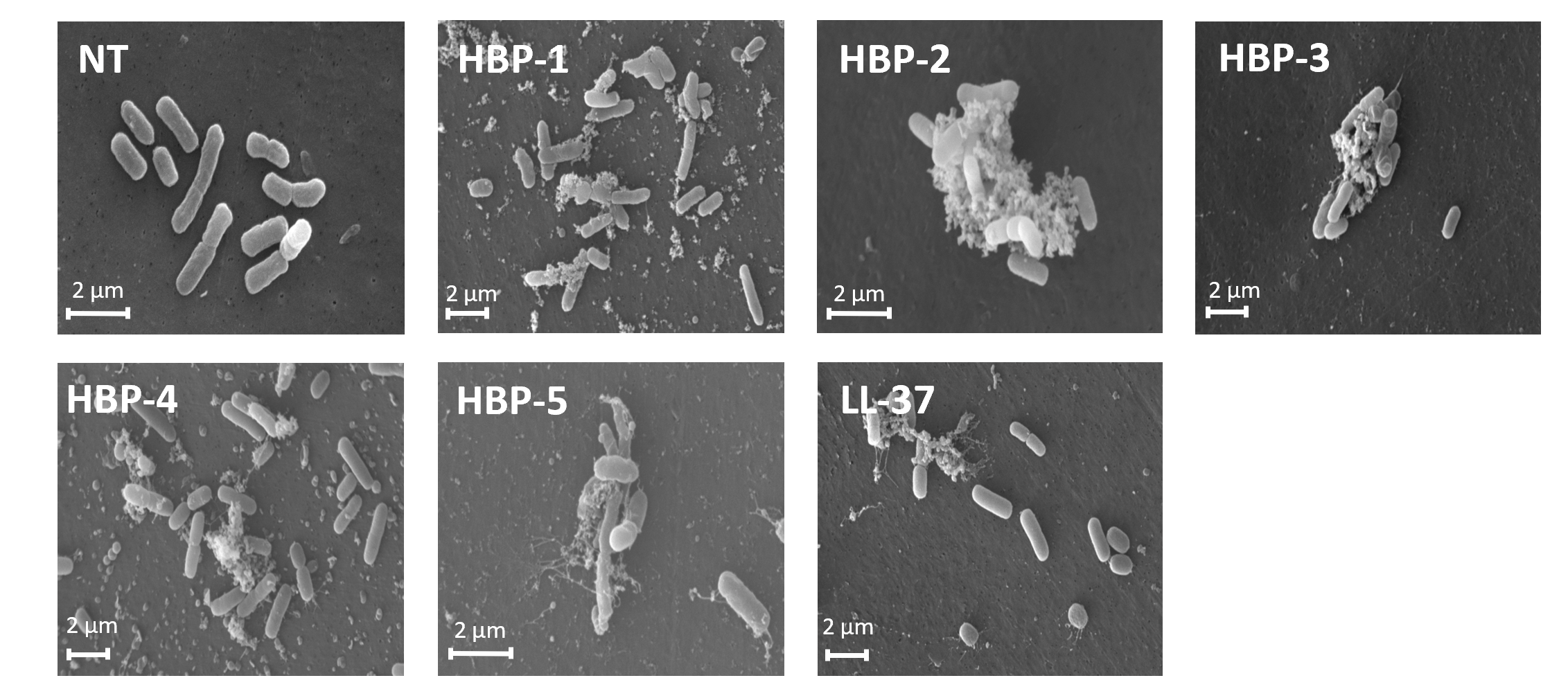

Supplement: Supplementary file 4 — Source data Fig. 2 [file 44320_2025_120_MOESM4_ESM.zip › SD figure 2/SEM images/2E.tif]
