# Supplementary material for: Mining the heparinome for cryptic antimicrobial peptides that selectively kill Gram-negative bacteria
Source: Mol Syst Biol. 2025 May 23;21(7):889–910. doi: 10.1038/s44320-025-00120-6 (PMC12223310; doi:10.1038/s44320-025-00120-6)
Supplement: Supplementary file 9 — Expanded View Figures [file 44320_2025_120_MOESM9_ESM.pdf]

Expanded View Figures

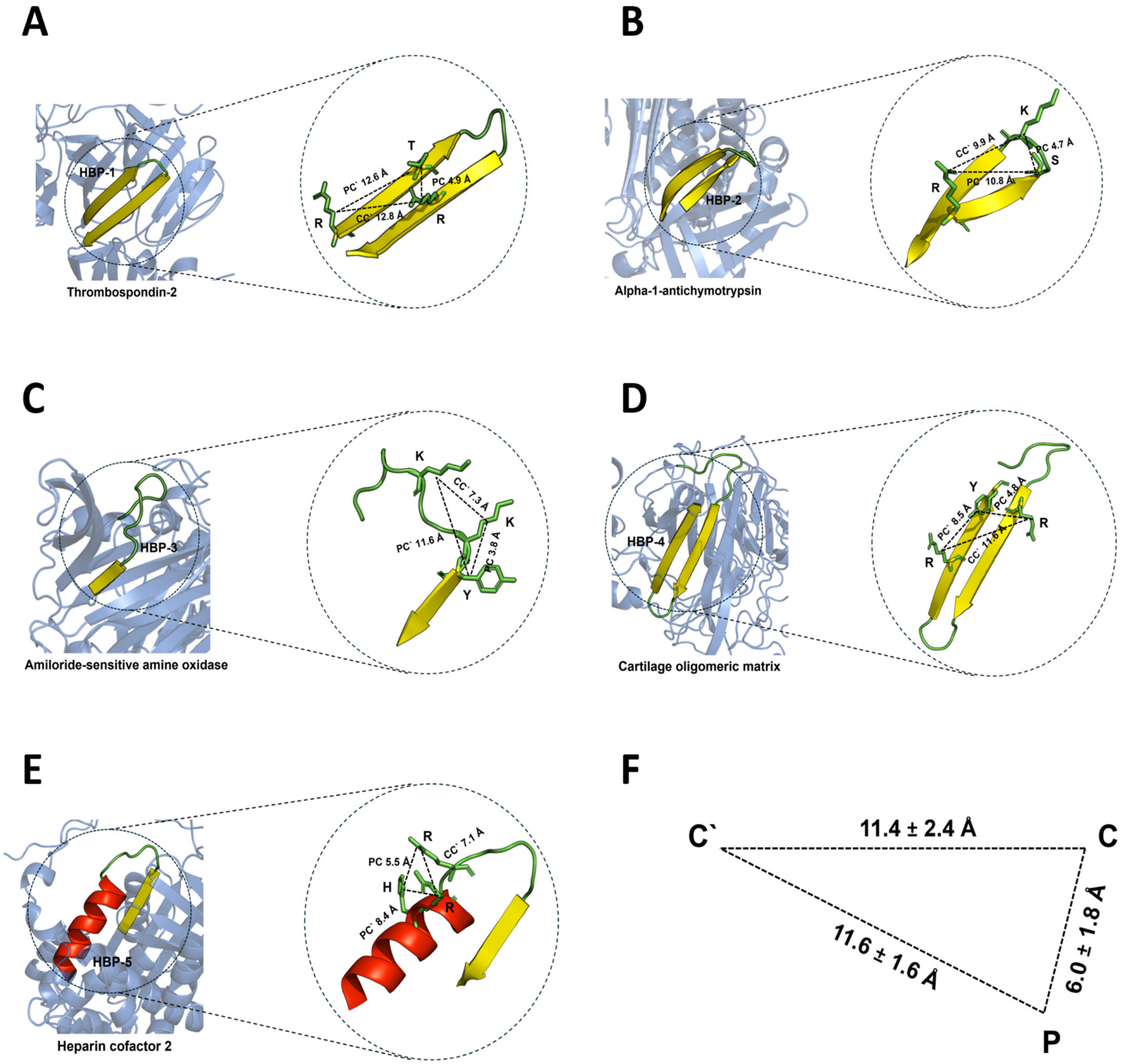

Figure EV1. The CPC′ clip motif of HBPs.

Three-dimensional structure of the five selected HBPs and their respective CPC motifs in the context of the parent heparin-binding proteins: (**A**) HBP-1, (**B**) HBP-2, (**C**) HBP-3, (**D**) HBP-4, (**E**) HBP-5 and, (**F**) An outline of the CPC′ clip motif. Tertiary structures are generated with Pymol. In the (**A–E**) CPC motifs helices are shown in red, β-strand in yellow, and loops are in green; remaining protein structures in transparent marine blue. The residues involved in the CPC′ clip are connected with dashed lines.

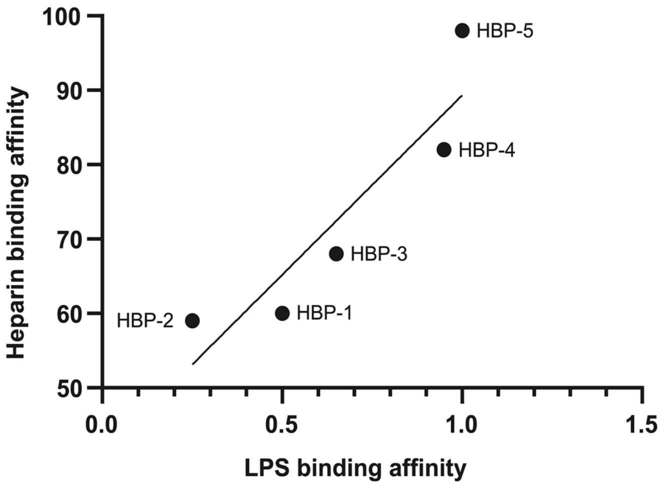

Figure EV2. Correlation between heparin and LPS-binding affinities of HBPs 1–5.

Heparin affinity measured as the % of elution buffer required to dislodge the peptides from a heparin column; LPS affinity is measured as EC_{50} values from the BODIPY-cadaverine assay. Source data are available online for this figure.

 © The Author(s)

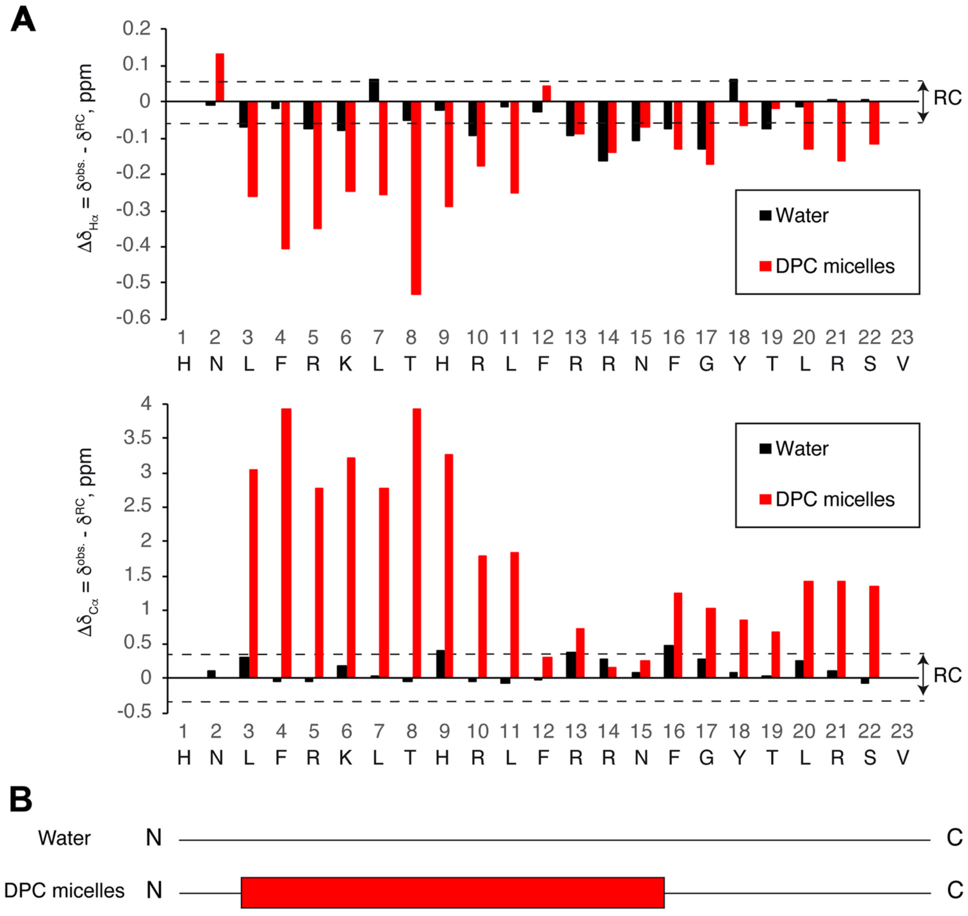

Figure EV3. NMR chemical shifts.

(**A**) $\Delta\delta_{H\alpha}$ and $\Delta\delta_{C\alpha}$ conformational shifts for HBP-5 in aqueous solution (black bars) and in DPC micelles (red bars) at pH 5.5 and 25 °C plotted as a function of peptide sequence. The two dashed lines indicate the random coil range (RC). (**B**) Schematic representation of the structural features in aqueous solution and in DPC micelles. Helices are shown as red rectangles. Source data are available online for this figure.

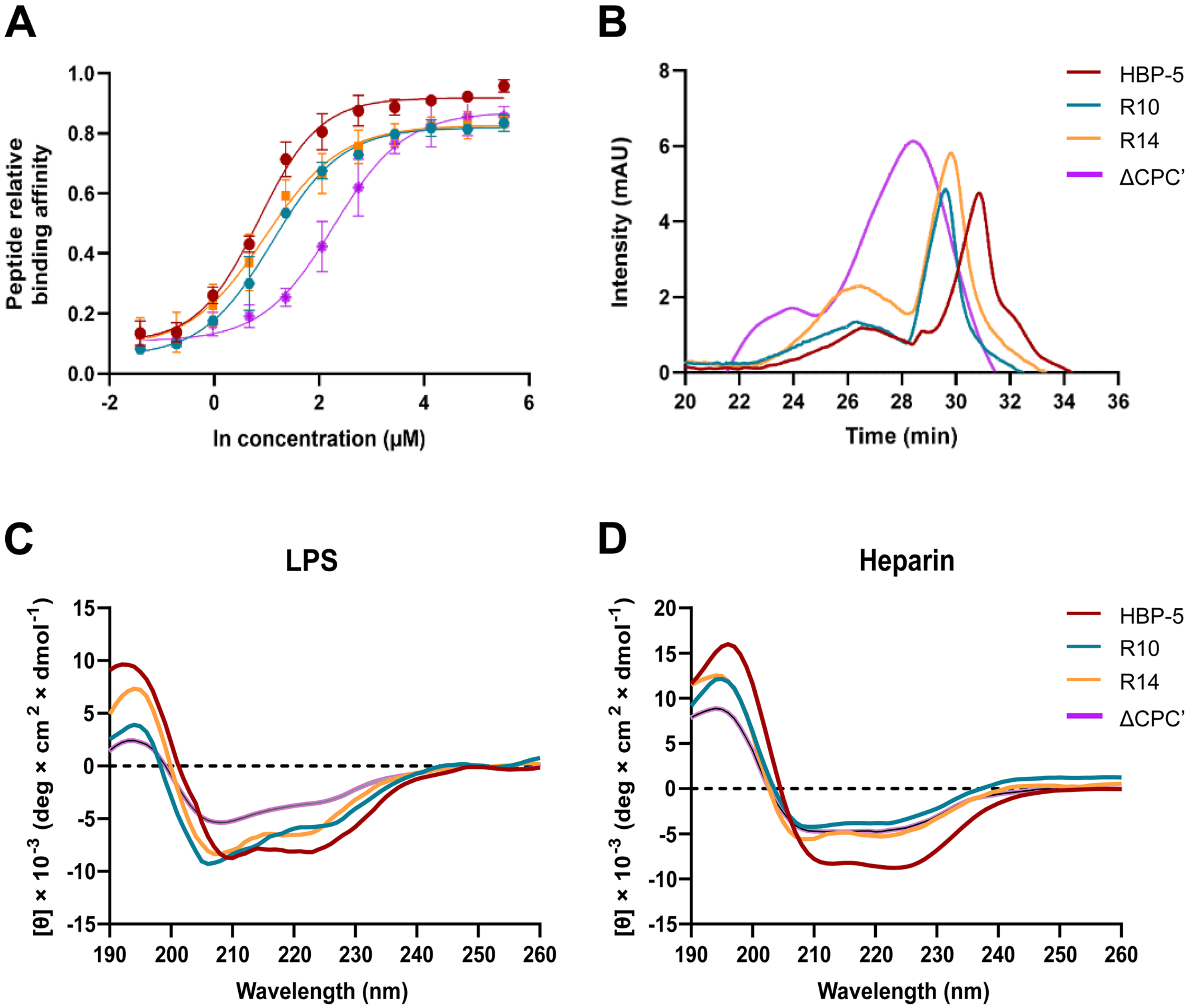

Figure EV4. Structure-activity relationship study of HBP-5 and CPC′ mutants.

(**A**) LPS affinity measured as increase in fluorescence emission (λ_{em} = 620 nm) of BODIPY-cadaverine at different peptide concentrations. (**B**) Heparin-binding affinities in FPLC chromatography. (**C**, **D**) CD spectra of peptides in 50 μg/mL LPS (left) and 20 μg/mL heparin (right). Source data are available online for this figure.

 © The Author(s)

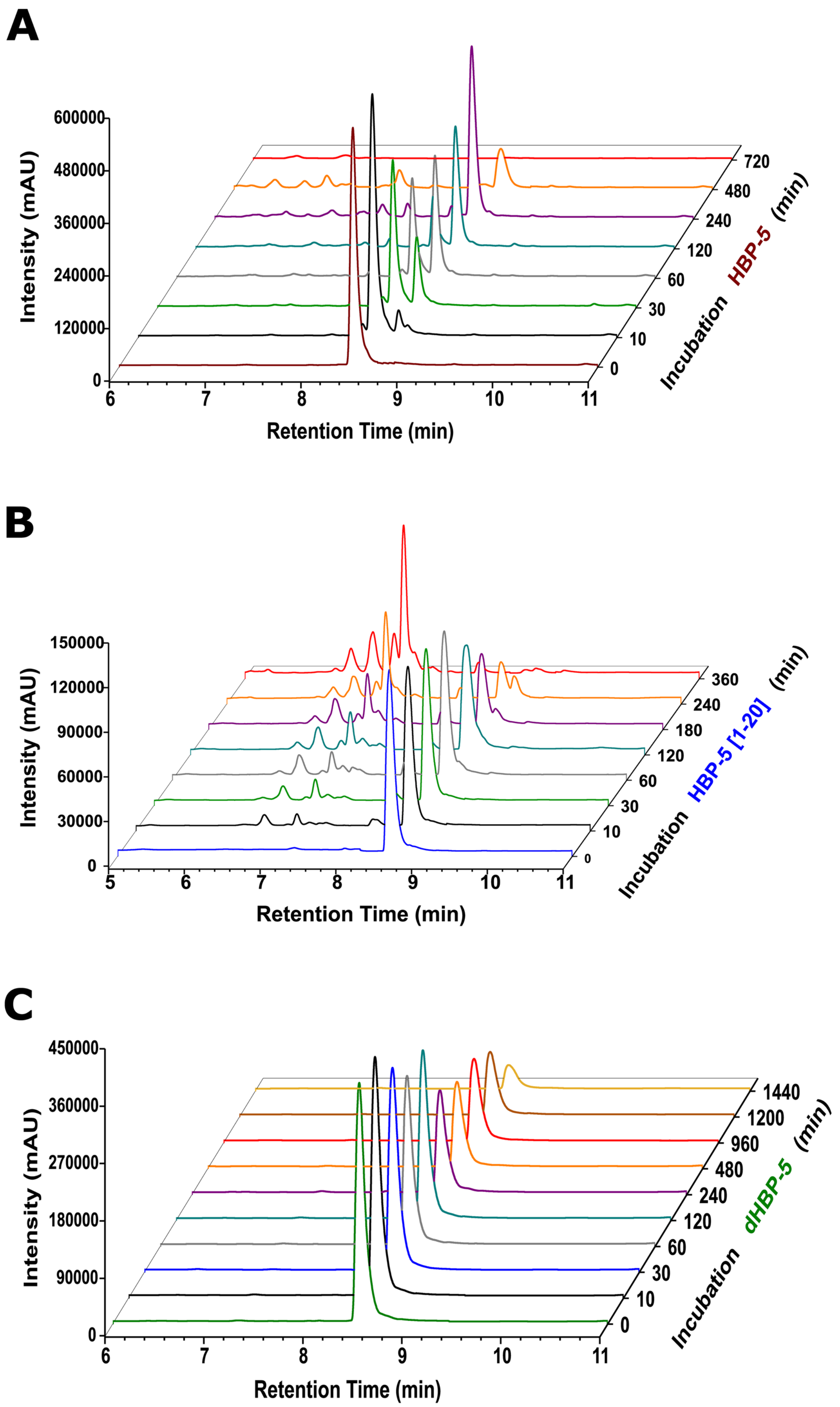
A
Intensity (mAU)
600000
480000
360000
240000
120000
0
6 7 8 9 10 11
Retention Time (min)
720 480 240 120 60 30 10 0
Incubation HBP-5 (min)
B
Intensity (mAU)
150000
120000
90000
60000
30000
0
5 6 7 8 9 10 11
Retention Time (min)
360 240 180 120 60 30 10 0
Incubation HBP-5 [1-20] (min)
C
Intensity (mAU)
450000
360000
270000
180000
90000
0
6 7 8 9 10 11
Retention Time (min)
1440 1200 960 480 240 120 60 30 10 0
Incubation dHBP-5 (min)

Figure EV5. RP-HPLC chromatograms of peptides in human serum.

Elution profiles after incubation of HBP-5 (**A**), HBP-5 [1–20] (**B**) and dHBP-5 (**C**) with 50% (v/v) human serum at representative times, using a 0% to 95% ACN gradient over 15 min. The peaks at t_0 correspond to the intact peptide. Source data are available online for this figure.

 © The Author(s)
